# FEN1 promotes tumor progression and confers cisplatin resistance in non‐small‐cell lung cancer

**DOI:** 10.1002/1878-0261.12058

**Published:** 2017-05-12

**Authors:** Lingfeng He, Libo Luo, Hong Zhu, Huan Yang, Yilan Zhang, Huan Wu, Hongfang Sun, Feng Jiang, Chandra S. Kathera, Lingjie Liu, Ziheng Zhuang, Haoyan Chen, Feiyan Pan, Zhigang Hu, Jing Zhang, Zhigang Guo

**Affiliations:** ^1^ Jiangsu Key Laboratory for Molecular and Medical Biotechnology College of Life Sciences Nanjing Normal University China; ^2^ Changzhou No. 7 People's Hospital China; ^3^ Department of Thoracic Surgery Jiangsu Cancer Hospital Affiliated Cancer Hospital of Nanjing Medical University China; ^4^ Southern University of Science and Technology of China Shenzhen China; ^5^ School of Pharmaceutical Engineering and Life Sciences Changzhou University China; ^6^ Division of Gastroenterology and Hepatology RenJi Hospital School of Medicine Shanghai Jiao Tong University China

**Keywords:** cisplatin resistance, flap endonuclease 1, lung cancer, targeted therapy

## Abstract

Lung cancer is one of the leading causes of cancer mortality worldwide. The therapeutic effect of chemotherapy is limited due to the resistance of cancer cells, which remains a challenge in cancer therapeutics. In this work, we found that flap endonuclease 1 (FEN1) is overexpressed in lung cancer cells. FEN1 is a major component of the base excision repair pathway for DNA repair systems and plays important roles in maintaining genomic stability through DNA replication and repair. We showed that FEN1 is critical for the rapid proliferation of lung cancer cells. Suppression of FEN1 resulted in decreased DNA replication and accumulation of DNA damage, which subsequently induced apoptosis. Manipulating the amount of FEN1 altered the response of lung cancer cells to chemotherapeutic drugs. A small‐molecule inhibitor (C20) was used to target FEN1 and this enhanced the therapeutic effect of cisplatin. The FEN1 inhibitor significantly suppressed cell proliferation and induced DNA damage in lung cancer cells. In mouse models, the FEN1 inhibitor sensitized lung cancer cells to a DNA damage‐inducing agent and efficiently suppressed cancer progression in combination with cisplatin treatment. Our study suggests that targeting FEN1 may be a novel and efficient strategy for a tumor‐targeting therapy for lung cancer.

**Abbreviations**
BER: base excision repair
DSB: double‐strand breaks
FEN1: flap endonuclease 1
HR: homologous recombination
IHC: immunohistochemistry
LP‐BER: long‐patch base excision repair
MMR: mismatch repair
NER: nucleotide excision repair
NHEJ: nonhomologous end‐joining
NSCLC: non‐small‐cell lung cancer
XPG: xeroderma pigmentosum complementation group G

## 1 Introduction

Lung cancer is a leading cause of global cancer‐related deaths for both men and women (Centers for Disease Control and Prevention, 2016). Non‐small‐cell lung carcinoma (NSCLC) accounts for approximately 85% of the lung cancer cases. Adenocarcinoma is the most common type of NSCLC that both smokers and nonsmokers suffer from. Nowadays in clinical therapy, most anticancer agents kill cells by interfering with DNA replication or by inducing DNA damage, which in turn leads to cell apoptosis (Gottesman, 2002; Liu, 2009). Among these anticancer drugs, cisplatin represents a successful landmark in the history of cancer clinical therapy. Once taken into the cells, cisplatin intercalates and forms intrastrand crosslinks in DNA, interferes with DNA replication and induces DNA damage, and eventually triggers apoptosis or necrosis. Cisplatin‐based doublets are widely used for NSCLC treatment and improve survival rates compared to placebo treatment (Rajeswaran *et al*., 2008). However, the efficacy of cisplatin is not adequate due to the highly effective DNA replication and repair system in cancer cells. The mechanisms of cisplatin resistance remain to be further revealed. Previous reports have suggested that cancer resistance to DNA damage‐inducing agents is associated with the elevated expression of DNA repair enzymes in cancer cells. For example, Lawson *et al*. have shown that DNA polymerase β, a DNA repair enzyme in base excision repair (BER), is overexpressed and determines etoposide resistance in small‐cell lung cancer (Lawson *et al.,* 2011). Liu *et al*. (2009) demonstrated that acquired resistance of cancer cells to chemotherapy is mediated by both BER and the homologous recombination (HR) of DNA repair pathways. Based on these reports, we hypothesized that the suppression of DNA repair enzymes in cancer cells could overcome cisplatin resistance of cancer cells.

DNA flap endonuclease 1 (FEN1) has been reported to be a key player in various DNA repair pathways. For example, in BER (Shen *et al*., 2005), FEN1 is involved in the removal of flap structures formed during long patch (LP). And in HR (Fehrmann *et al*., 2015; Kikuchi *et al*., 2005) and mismatch repair (Liu *et al*., 2015), FEN1 is also involved. Besides, FEN1 has been shown to be involved in nucleotide excision repair (NER) by associating with ligase I in the final step of NER (Mocquet *et al*., 2008). Moreover, FEN1 and the NER protein XPG (xeroderma pigmentosum complementation group G) show homology in the DNA‐binding domain, suggesting that FEN1 may support XPG function in NER (Herrero *et al*., 2006). Given that the cisplatin‐induced intrastrand crosslink of DNA adduct was mainly repaired by NER, we hypothesized that the suppression of FEN1 expression or inhibition of FEN1 activity might augment the therapeutic response of cisplatin. FEN1 was initially reported to play a role in DNA replication by removing the RNA primer during Okazaki fragment maturation of the lagging strand (Balakrishnan and Bambara, 2013; Klungland and Lindahl, 1997). Consistent with its function in DNA replication, FEN1 is required to support the hyperproliferation of cells. Indeed, FEN1 is expressed at low levels in quiescent cells (Kim *et al*., 2000) but is highly expressed in proliferative tissues and cancers, including lung (Nikolova *et al*., 2009), breast (Singh *et al*., 2008), gastric (Wang *et al*., 2014), prostate (Lam *et al*., 2006), pancreatic (Iacobuzio‐Donahue *et al*., 2003), and brain cancers (Krause *et al*., 2005). The level of FEN1 expression in cancerous tissues has been correlated with advanced cancer grade and aggressiveness (Abdel‐Fatah *et al*., 2014; Lam *et al*., 2006).

In view of the role of FEN1 in DNA replication, we speculated that FEN1 might be essential for cell proliferation of lung cancers. The fact that FEN1 is involved in NER and other DNA repair pathways prompted us to further speculate that targeting FEN1 could be a potential way to overcome the drug resistance of lung cancer to cisplatin. FEN1 inhibitor could be used as a stand‐alone agent for blocking cancer cell proliferation or combining with DNA damage‐inducing agents to augment the therapeutic efficacy. By using the A549 cell line as a research model, we demonstrated that FEN1 was essential for proliferation and cisplatin resistance of lung cancer cells. Inhibition of FEN1 suppressed cell growth and resulted in the accumulation of DNA double‐strand breaks, thereby inducing apoptosis. Furthermore, FEN1 inhibitor impeded the progression of lung cancer and resulted in an accumulative effect when combined with cisplatin *in vitro* and on xenograft tumor mice models. Our work showed that targeting FEN1 could be a potential strategy for lung cancer therapy.

## 2 Materials and methods

### 2.1 Cell lines and cell culture

The human lung cancer cell lines A549, H1299, and H460 were obtained from ATCC (Manassas, VA, USA). These cells were cultured under conditions described by the products' instructions. The human embryonic lung fibroblast cell line HELF was cultured in DMEM (Invitrogen, Carlsbad, CA, USA) supplemented with 10% fetal bovine serum (FBS).

### 2.2 Antibody

Antibodies used in this paper are listed here: anti‐P53 antibody (SC‐126; Santa Cruz Biotechnology, Inc., Dallas, TX, USA), anticaspase‐3 (SC‐7148; Santa Cruz Biotechnology, Inc.), antivinculin antibody (MAB3574; Millipore, Bedford, MA, USA), anti‐FEN1 (70185; GeneTex, Inc., Irvine, CA, USA), antitubulin (AM103a; Bio‐world, Dublin, OH, USA), anti‐GAPDH (AP0063; Abgent, Suzhou, China), anti‐γ‐H2AX (ab2893; Abcam, Cambridge, MA, USA), anticleaved caspase‐3 antibody: (Asp175) antibody #9661 (Cell Signaling Technology, Danvers, MA, USA), antiphospho‐P53: phospho‐p53 (Ser15) antibody #9284 (Cell Signaling Technology), anti‐Myc‐tag (AP0031M; Abgent), P53BP1 (SC‐22760; Santa Cruz), Alexa Fluor ^®^488 goat anti‐rabbit A‐11008 Life Technologies, Alexa Fluor ^®^594 donkey anti‐rabbit R37119 Life Technologies.

### 2.3 Antitumor effect on tumor xenografts in nude mice

All animal experiments were conducted in accordance with the National Institutes of Health Guide for the Care and Use of Laboratory Animals. Male 4‐ to 5‐week‐old BALB/C nude mice were used in this study. A549 cells (2 × 10^6^) suspended in 100 μL serum‐free medium were inoculated subcutaneously. Approximately two weeks later, when the average tumor volume reached 100–120 mm^3^, the mice were randomly divided into groups. FEN1 inhibitor (10 mg·kg^−1^ mice body weight) and cisplatin (2 mg·kg^−1^ mice body weight) were administered intraperitoneally daily for five consecutive days. Tumor sizes were measured by a Vernier caliper every week thereafter, and tumor volumes (mm^3^) were calculated as length × width^2^/2. All mice were euthanized when the cancer volumes in the control mice reached ∼ 1000 mm^3^. The mice were housed and maintained under standard NIH protocol.

### 2.4 Immunofluorescence staining

Cells were cultured in six‐well plates containing acid‐treated cover slides and incubated overnight. The cover slides were then washed with PBS, fixed with 4% formaldehyde in PBS for 30 min, and washed with PBS again. Triton X‐100 (0.05%) was added for 5 min to permeabilize the cells. Slides were blocked with 3% BSA and then incubated with primary antibody. The slides were washed, incubated with secondary antibody conjugated with FITC, washed again with PBS, and stained with DAPI. The mounted slides were viewed with a Nikon 80I 10‐1500X microscope, and images were captured with a camera.

### 2.5 Flow cytometric analysis

Cells were trypsinized, washed, and resuspended in 1 mL PBS with 5% FBS. Subsequently, cells were washed twice with ice‐cold PBS and fixed with 3 mL ice‐cold ethanol. After centrifugation, cells were resuspended with 1 mL 50 μg·mL^−1^ RNase A and 50 μg·mL^−1^ propidium iodide (PI) at 37 °C for 30 min. The apoptosis ratio was then analyzed using a FACS flow cytometer (Calibur, BD Biosciences, San Jose, CA, USA).

### 2.6 TUNEL (TdT‐mediated dUTP Nick‐End Labeling) assay

Cells were cultured in six‐well plates containing acid‐treated cover slides and incubated overnight. The cover slides were then washed with PBS, fixed with 4% formaldehyde in PBS for 30 min, and washed with PBS again. Triton X‐100 (1%) was added for 5 min to permeabilize the cells. Three percent H_2_O_2_ was then added for 10 min and cover slides were washed twice with ice‐cold PBS. Cells were incubated with TdT marker solution at 37 °C for 1 h and then gently washed with PBS three times. Cells were incubated in the dark with 100 μL dyeing buffer solution for 30 min, washed with PBS, and stained with DAPI.

### 2.7 Metaphase spread preparation

Cells were collected and treated with colchicine to arrest cells at metaphase. Cells were incubated (20 min, room temperature) with hypotonic solution (75 mm KCl), placed in a 37 °C water bath (5 min), and fixed with Carnoy's solution. The fixation process was repeated three times, and a dropper was used to place cells onto a clean slide. The cell spread was incubated (55 °C overnight), stained with Giemsa solution, and checked for aberrant chromosomes under a microscope.

### 2.8 Colony‐forming assay

Cells were plated in 6‐cm dishes and incubated for approximately 15 days at 37 °C. The cells were then washed with PBS and stained with 0.05% crystal violet. Stained plates were washed and dried prior to scoring the colonies.

### 2.9 Lentivirus and stable cell line preparation

Lentivirus particles expressing the FEN1 gene were generated by transfecting 293T cells with the FEN1 plasmids together with packaging plasmids. The virus‐containing medium was collected every 24 h for 3 days. The cells were incubated with the lentivirus‐containing medium plus 4 μg·mL^−1^ polybrene for 24 h and were then selected after 72 h in 1.0 μg·mL^−1^ puromycin. All lentivirus particles were prepared by Guangzhou Fitgene Biotechnology Co., Ltd., Guangzhou, China.

### 2.10 Immunocytochemistry analysis

Tissues were fixed in 10% formalin. Paraffin‐embedded sections from tissue specimens were deparaffinized and heated at 97 °C in 10 mm citrate buffer (pH 6.0) for 20 min for antigen retrieval. Primary antibodies used in immunocytochemistry were raised against FEN1. Immunoreactivities were analyzed by estimating the percentage of cells showing characteristic staining and the intensity of staining. The intensity of staining was graded as 1 (weak), 2 (medium), or 3 (strong). The results were scored by multiplying the percentage of positive cells (P) by the intensity (I) to obtain the Q‐score (Q), which ranged between 0 and 300. A Q‐score of 300 indicated that 100% of the cells were strongly stained (Q = P × I; maximum = 300).

### 2.11 Drug sensitivity assay

Sensitivity to a DNA damage reagent was determined by a cell growth inhibition assay (Simpson *et al*., 2010). A549 cells were seeded (1500 per well), incubated (overnight, 37 °C), treated (1 h, 37 °C) with multiple dilutions of H_2_O_2_, washed in a fresh medium (DMEM containing 10% FBS), and incubated (72 h) under normal growth conditions (37 °C, 5% CO_2_). The number of viable cells was determined by the CellTiter 96 AQueous one‐solution cell proliferation assay (Promega, Madison, WI, USA). At least four replications for each clone were averaged. Data were expressed as the percentage of growth relative to untreated controls.

## 3 Results

### 3.1 FEN1 was up‐regulated in lung cancer cells and associated with poor prognosis

**Figure 1:**
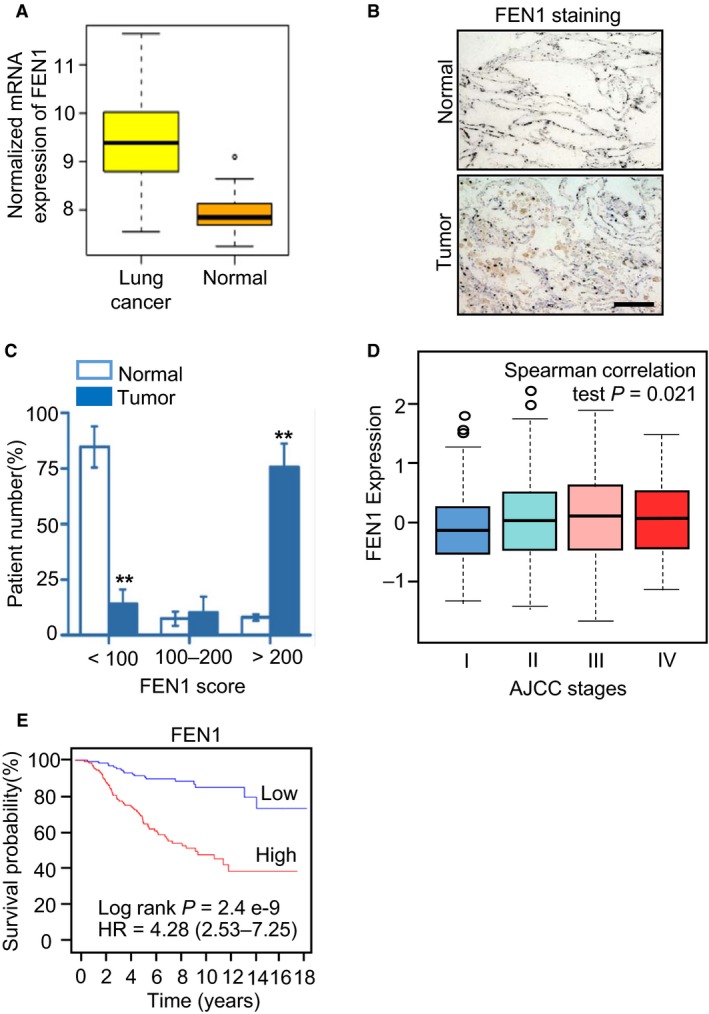
FEN1 overexpression was associated with lung cancer. (A) FEN1 expression analysis based on TCGA dataset showed that FEN1 mRNA levels were higher in lung cancer tissue than in normal tissue (*P < 0.01 vs control group). (B) FEN1 displayed significantly stronger staining (brown) in tumor specimens from clinical patients than from healthy counterparts. Immunohistochemistry was performed on formalin‐fixed and paraffin‐embedded tissues using antibodies against FEN1. Original magnification, × 400. Scale bars, 250 μm. (C). Number of patients' samples with FEN1 score > 200 was significantly higher in tumors than in normal tissues (**P < 0.01 vs control group). (D) FEN1 expression was correlated with the clinical stage of lung cancer. Spearman's correlation test, *P* = 0.021. (E) Kaplan–Meier analysis of survival of patients with lung cancer. Log rank *P* = 2.4e‐9. (F) FEN1 protein was elevated in cultivated NSCLC cells compared to normal cells. The bottom panel was the quantification results of the top panel.

As an essential player in DNA replication, FEN1 is expected to be up‐regulated in cancerous tissues. To verify this hypothesis, we searched the TCGA database and compared the FEN1 expression levels between cancerous and normal tissues. The results showed that FEN1 mRNA expression level in lung cancer tissues was significantly higher than that in normal tissues (Fig. 1A). We confirmed this observation by immunohistochemistry (IHC) assays comparing the FEN1 protein expression level on normal and lung cancer samples from surgical treatment (Fig. 1B). In normal tissue, the proportion of patients with a FEN1 score below 100 was 85% (95% CI, 76–97, *P* < 0.01). This proportion was significantly lower in cancer tissues (12%, 95% CI, 8–15, *P* < 0.01). Conversely, the proportion of patients with FEN1 scores above 200 was significantly higher in cancerous tissues than in normal ones (76% vs 12%, *P* < 0.001, Fig. 1C).

Next, we investigated whether the FEN1 expression level was associated with the malignancy of lung cancers. Data from the TCGA database indicated that malignancy grade rose with increasing FEN1 expression levels in lung cancers (Fig. 1D and Table S1), suggesting that the malignancy of lung cancer was correlated with FEN1 overexpression. In support of this correlation, patients with high levels of FEN1 had significantly shorter overall survival time than those with low levels of FEN1 (Fig. 1E). These results suggest that FEN1 was up‐regulated in lung cancers, associated with tumor malignancy and poor prognosis.

### 3.2 FEN1 promoted tumor progression *in vitro* and *in vivo*

**Figure 2:**
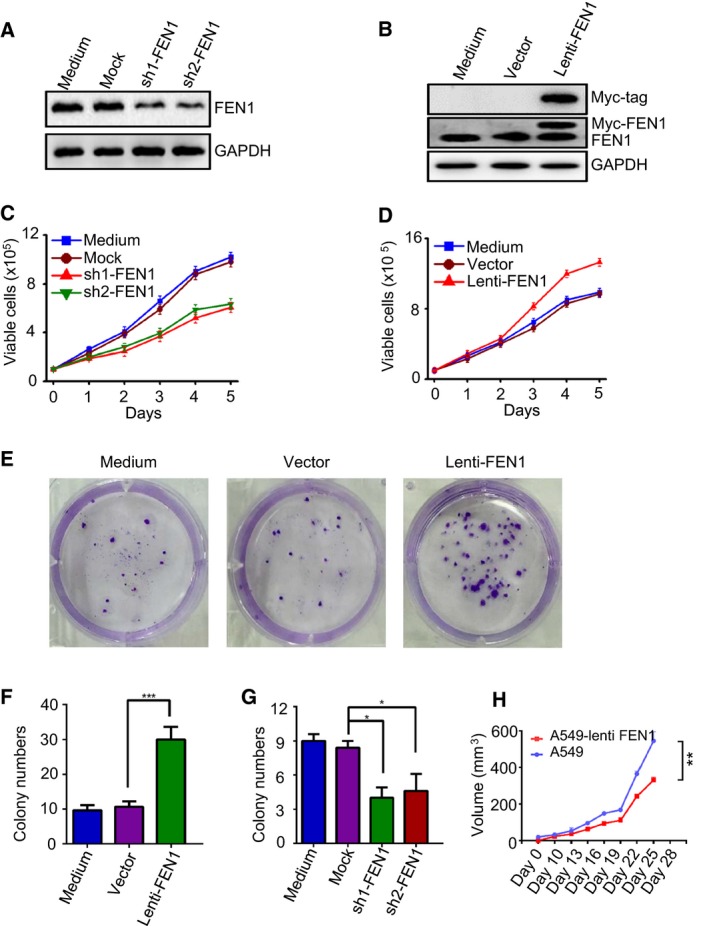
FEN1 facilitated tumor progression. (A) Generation of cell lines with FEN1 knockdown. (B) Establishment of cell lines with ectopic overexpression of FEN1. In panels A and B, the FEN1 levels in cells were determined by western blotting using anti‐FEN1 antibody. (C) FEN1 knockdown decreased the growth of A549 cells. Data represent mean ± SD from three independent assays. (D) Overexpression of FEN1 promoted the growth of A549 cells. Data represent mean ± SD from three independent assays. (E) Representative image for colony‐forming assay. Panel E showed that FEN1 overexpression enhanced colony formation of A549 cells. (F) A statistic of the clone number for panel E (error bars represent SEM, and statistical significance was determined by paired *t*‐test. ***P < 0.001. Data were compared between untreated control with respect to treatment, ***P < 0.001). (G). FEN1 knockdown reduced the colony formation efficiency in A549 cells (*P < 0.05). (H). A549 cells with FEN1 overexpression resulted in increased tumor growth and volume compared with parental A549 cells.

The observation that FEN1 was up‐regulated in lung cancers was further confirmed in cancer cell lines. As shown in Fig. S3, lung cancer cell lines (A549, H1299, and H460) displayed significantly higher FEN1 expression level than the normal lung cell line (HELF). A549 cells were chosen in this study because they were widely used as an *in vitro* model for NSCLC drug metabolism research. The data above have already indicated that FEN1 overexpression was associated with cancer. But whether high expression level of FEN1 would influence tumor progression was still not clear. We speculated that FEN1 promoted tumor progression. To test this hypothesis, we compared the proliferation rate of A549 cells in which FEN1 was down‐regulated by siRNA (Fig. 2A) with those in which FEN1 was ectopically overexpressed (Fig. 2B). The results showed that the down‐regulation of FEN1 suppressed cell growth (Fig. 2C), whereas overexpression of FEN1 promoted cell growth (Fig. 2D). Moreover, the colony‐forming assay showed that overexpression of FEN1 induced colony formation (Fig. 2E–F), while the down‐regulation of endogenous FEN1 reduced colony formation efficiency in A549 cells (Fig. 2G). In addition, flow cytometry analysis showed that FEN1 knockdowns led to a decrease in S and G2/M phase proportions and an increase in G1 proportions compared to control cells (data not shown). Furthermore, after being transplanted subcutaneously into nude mice, cells with ectopic FEN1 overexpression gave rise to significantly bigger tumors than parental A549 cells did (Fig. 2H). Under the same conditions, the transplantation of A549 cells with FEN1 knockdown into nude mice did not lead to tumor formation (data not shown). These data verified our speculation that FEN1 promoted tumor progression *in vitro* and *in vivo*.

### 3.3 FEN1 contributed to cisplatin resistance of A549 cells

**Figure 3:**
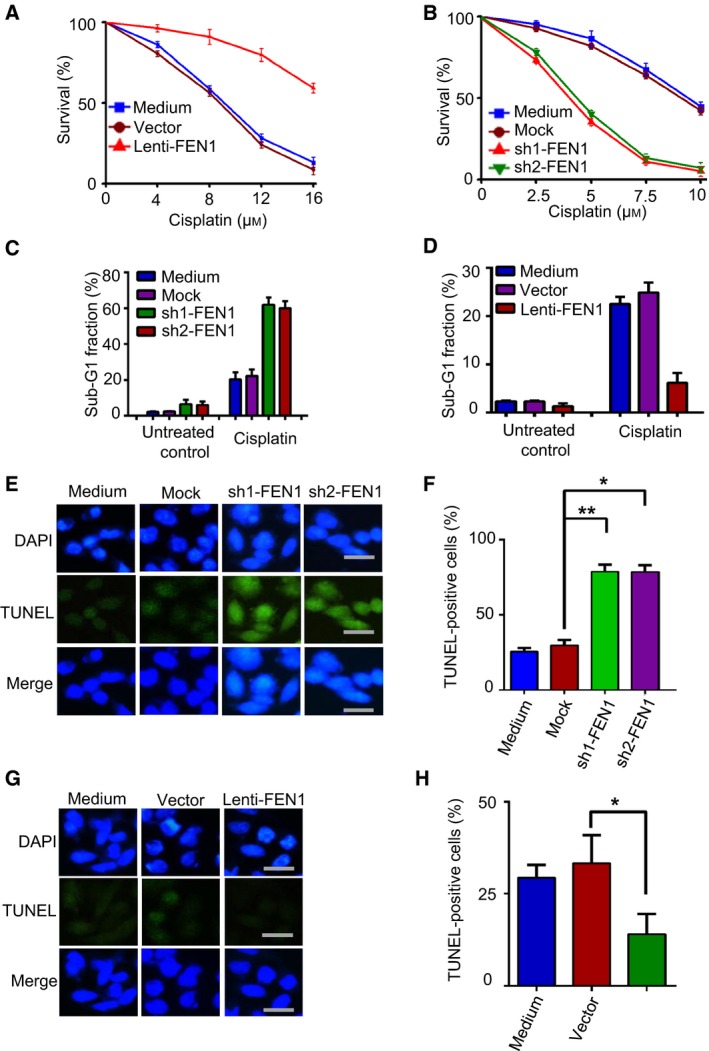
FEN1 contributed to cisplatin resistance of lung cancer cells. (A). FEN1 overexpression enhanced the resistance of A549 cells to cisplatin. Data represent mean ± SD from three independent assays. (B) Knockdown of FEN1 sensitized A549 cells to cisplatin. Data represent mean ± SD from three independent assays. (C) FACS analysis showed that cisplatin induced more sub‐G1 portion in FEN1‐knockdown cells than in control cells. Error bars represent SEM, and statistical significance was determined by paired *t*‐test. Data were compared between untreated controls with respect to treatment. *P < 0.05; **P < 0.01; ***P < 0.001. (D) FACS analysis showed that FEN1 overexpression in cells reduced cisplatin‐induced sub‐G1. (E) TUNEL assay indicated that cisplatin‐induced apoptosis was significantly higher in FEN1‐knockdown cells than in control cells. Shown are the representative images. Scale bars, 50 μm. (F) A statistic of the apoptotic number for panel E (**P < 0.01, *P < 0.05). (G). TUNEL assay indicated that FEN1 overexpression could reduce cisplatin‐induced apoptosis. Shown are the representative images. Scale bars, 50 μm. (H) A statistic of the apoptotic number for panel G (*P < 0.05).

Cisplatin is thought to enter the tumor cells, causing various types of DNA damage and triggering apoptosis/necrosis. Sensitivity to cisplatin, therefore, was predicted to be mediated by DNA repair pathways (Rosell *et al*., 2002). In view of the roles of FEN1 in multiple repair processes, such as BER (Simpson *et al*., 2010), nonhomologous end‐joining (Cao *et al*., 2016), HR (Hu *et al*., 2016; Liu *et al*., 2016b), NER (Herrero *et al*., 2006; Mocquet *et al*., 2008; Zhao *et al*., 2016), and mismatch repair (MMR; Liu *et al*., 2016a), it was pertinent to speculate that the high level of FEN1 expression in tumors contributed to intrinsic or acquired drug resistance. To test this hypothesis, we performed drug‐sensitive experiments in A549 lung cancer cells with different FEN1 levels. The results showed that the overexpression of FEN1 served as a protective effect against cisplatin treatment (Fig. 3A). At the same time, FEN1 knockdown sensitized A549 cells to cisplatin (Fig. 3B). To further analyze the impact of FEN1 on cell death, the sub‐G1 fraction was determined after cisplatin treatment. The results showed that knockdown of FEN1 resulted in the accumulation of sub‐G1 fraction after cisplatin exposure (Fig. 3C). Overexpression of FEN1 reduced the generation of cisplatin‐induced sub‐G1 fraction (Fig. 3D). Consistently, TUNEL staining showed that FEN1‐knockdown cells were more apoptotic than control cells after cisplatin treatment (Fig. 3E,F), while FEN1‐overexpressing cells were less apoptotic than control cells (Fig. 3G,H). These data suggested that FEN1 was protecting the cells from cisplatin‐induced apoptosis.

We have developed drug‐resistant cell and designed experiments to evaluate the possibility to overcome cisplatin resistance. As shown in Fig. S4A, cisplatin‐resistant cell was established from A549 lung cancer cells and we name it as A549‐cisplatin‐R cells or A549‐R for abbreviation. The A549‐R cells can grow well in the medium with 2 μg·mL^−1^ cisplatin but wild‐type A549 cells died after 3 days' cultivation (Fig. S4B). To evaluate the possibility to overcome cisplatin resistance, we treated A549‐R cells with 10 and 20 μm FEN1 inhibitor C20 for 3 days. As shown in Fig. S4C, A549‐R cells partially lost the resistance to cisplatin.

### 3.4 FEN1 expression affected the repair of cisplatin‐induced DNA damage in A549 cells

**Figure 4:**
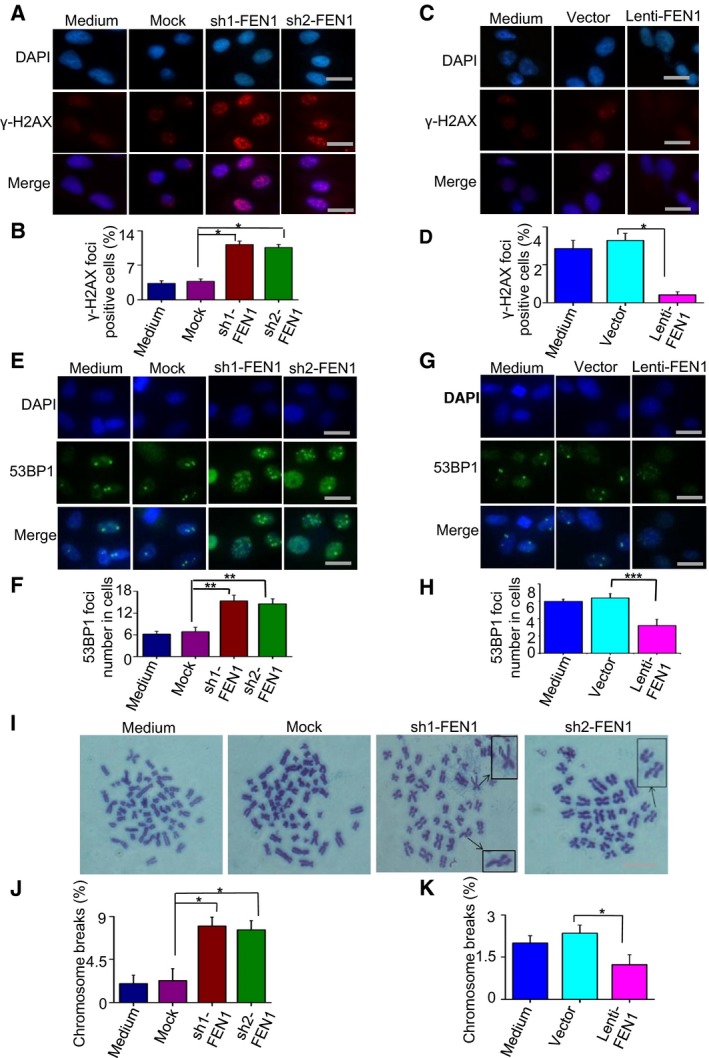
FEN1 contributed to the repair of cisplatin‐induced DNA damage. (A) Cell immunostaining assay showed that more γ‐H2AX foci formed in FEN1‐knockdown A549 cells after cisplatin treatment (5 μm, 72 h). Scale bars, 50 μm. (B) Quantification of panel A. (C) Cell immunostaining assay showed that FEN1 overexpression reduced cisplatin‐induced γ‐H2AX foci formation in A549 cells. Scale bars, 50 μm. (D) Quantification of panel C. Cell staining with 53BP1 antibody showed that cisplatin‐induced 53BP1 foci were increased by FEN1 knockdown (E) but decreased by FEN1 overexpression in A549 cells (G). (F) and (H) were the quantification data for panels E and G, respectively. Scale bars, 50 μm. (I) Representative image for chromosome aberrations assay. Chromosome in FEN1‐knockdown cells was more fragile after cisplatin exposure (5 μm, 72 h). Detail shows a magnification of chromosome aberration. (J) was the statistical quantification for panel I, and (K) was the statistical quantification of chromosome aberrations in FEN1 overexpression in A549 cells under treatment with cisplatin.

Repair defects of cisplatin‐induced damage can lead to the accumulation of unrepaired DNA intermediates and DNA double‐strand breaks (DSBs) (Guo *et al*., 2010, 2012; Zheng *et al*., 2007a, 2011). Therefore, we predicted that cells with FEN1 down‐regulation would show higher levels of DNA DSBs compared with control cells. To test this hypothesis, we determined the foci of γH2AX and 53BP1, markers of DNA DSBs in cells. Indeed, down‐regulation of FEN1 resulted in the accumulation of γH2AX (Fig. 4A,B) and 53BP1 (Fig. 4E,F) in cells. Conversely, FEN1 overexpression reduced the level of cisplatin‐induced foci formation of γH2AX (Fig. 4C,D) and 53BP1 (Fig. 4G,H) in cells.

Accumulation of unrepaired DNA damage induced by cisplatin will consequently cause chromosomal breaks (van Gent *et al*., 2001; Soza *et al*., 2009). To test the impacts of FEN1 on chromosomal breaks induced by cisplatin, we analyzed metaphase nuclei for chromosomal aberrations. FEN1‐deficient cells exhibited significantly increased levels of chromosomal fragments and breaks compared with the controlled parental ones (Fig. 4I–J). However, cells with high FEN1 expression level displayed reduced levels of chromosomal breakage (Fig. 4K).

### 3.5 FEN1 inhibitor resulted in the accumulation of unrepaired DSBs and enhanced sensitivity to cisplatin

Based on the results above, we inferred that a FEN1‐specific inhibitor might be able to serve as an anticancer drug which could be either used alone to suppress cancer cell growth or combined with DNA damage‐inducing agents to improve therapeutic efficacy. To test this hypothesis, a previously reported FEN1 inhibitor (compound 20 C20) was used (Exell *et al*., 2016; Tumey *et al*., 2005). Compound #20 (Fig. S2) is an N‐hydroxyl urea derivative that specifically inhibits FEN1 activity, with an IC50 of 3 nm, the most potent FEN1 inhibitor tested *in vitro* at the time (He *et al*., 2016).

**Figure 5:**
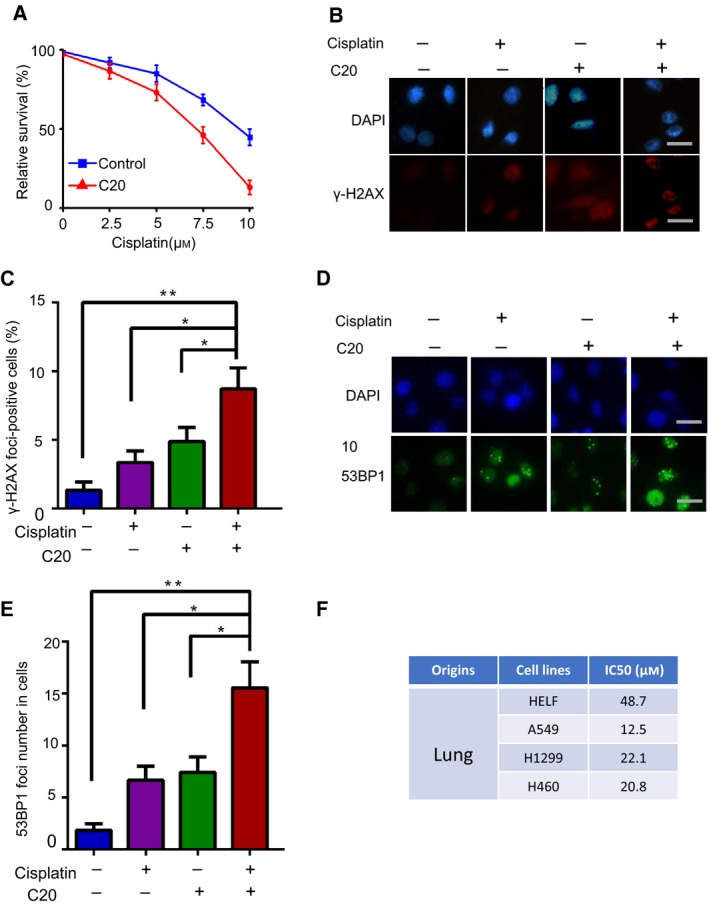
FEN1 inhibitor increased DSBs and enhanced sensitivity to cisplatin. (A). Pretreatment of cells with FEN1 inhibitor (8 μm, 48 h) enhanced cisplatin‐mediated growth inhibition in A549. FEN1 inhibitor resulted in the accumulation of unrepaired DSBs induced by cisplatin, as indicated by γ‐H2AX (B) or 53BP1 (D) staining. Data represent mean ± SD from three independent assays. Scale bars, 50 μm. (C) and (E) were the statistical quantification of panels B and D, respectively. (F). The IC50 of FEN1 inhibitor, C20, to various cells with different FEN1 levels. Data represent mean ± SD from three independent assays.

To determine whether targeting the inhibition of FEN1 enhanced the activity of cisplatin in A549 cells, A549 cells were pretreated with FEN1 inhibitor, followed by cisplatin treatment at various concentrations for 48 h. Figure 5A shows a significant decrease in the survival of C20‐treated cells. In support of these data, the cells with both C20 and cisplatin treatments showed a higher number of γH2AX and 53BP1 foci compared with the ones with cisplatin‐only treatment (Fig. 5B–E).

To evaluate the toxicity of FEN1 inhibitor on cells with different FEN1 levels, we compared the IC50 of C20 among various lung cancer cell lines. The results showed a dose‐dependent decrease in the cell proliferation of A549, H1299,and H460 (data not shown) with an IC50 of 12.5, 22.1, and 20.8 μm, respectively (Fig. 5F). Notably, cells with low FEN1 expression levels were less sensitive to FEN1 inhibitor. IC50 of HELF (48.7 μm) was much higher than that of A549 cells, indicating that the expression of FEN1 in different cancer cells could be correlated with their sensitivity to FEN1 inhibitor.

### 3.6 Antitumor effects of FEN1 inhibition on xenografts tumor mice

**Figure 6:**
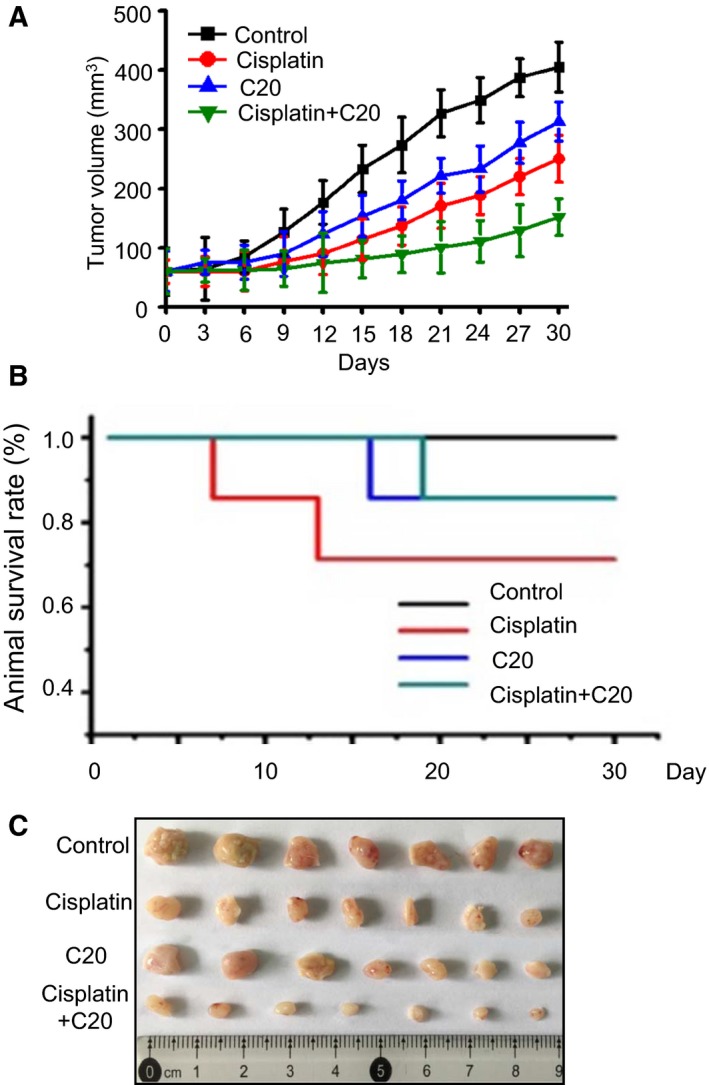
Antitumor effects of FEN1 inhibition on xenografts tumor mice. (A) Comparison of tumor progression induced by A549 cells after treatment with FEN1 inhibitor or combination with cisplatin. Animal models were prepared by injecting A549 cells subcutaneously into the right flank of nude mice. Error bars represent SEM, and statistical significance was determined by paired *t*‐test. ***P < 0.001. Data were compared between untreated controls with respect to treatment. (B) Animal survival rate was estimated by the Kaplan–Meier survival curves. (C) Tumor volume was monitored during cisplatin, C20 treatment, or a combination treatment.

To further investigate the impact of FEN1 inhibition on tumor progression *in vivo*, we used nude mice to do a xenograft study. The A549 cells were transplanted subcutaneously into nude mice. After the tumor volume reached 100–200 mm^3^, mice were treated with the FEN1 inhibitor, cisplatin, or the combination of the FEN1 inhibitor and cisplatin. The growth of tumors was monitored up to 30 days. As shown in Fig. 6A, the tumor volume gradually increased in control mice in a time‐dependent manner. Treatment with cisplatin or FEN1 inhibitor alone resulted in a slight decrease in the growth of xenograft tumors. When cisplatin was combined with the FEN1 inhibitor, the tumor growth was significantly reduced. At the same time, the animal survival rate also improved in the combined treatment group, as indicated in Fig. 6B. These results suggested that the inhibition of FEN1 could augment the efficacy of cisplatin in the lung cancer xenograft mouse model.

### 3.7 FEN1 down‐regulation or inhibition activated the intrinsic pathway of apoptosis

**Figure 7:**
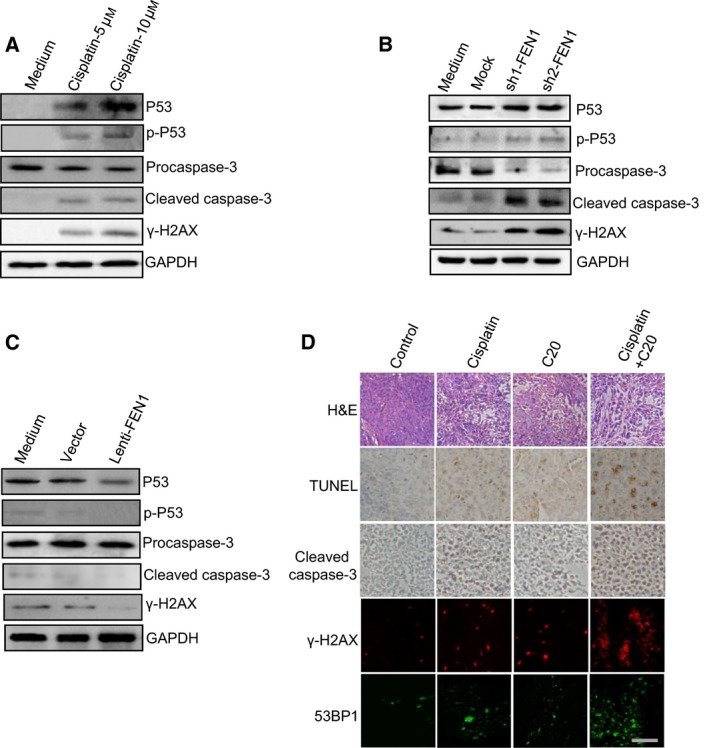
Mechanisms of the enhancement of cisplatin‐induced apoptosis by down‐regulating FEN1 in cancer cells. (A) Cisplatin treatment induced DNA damage and activated p53 and caspase‐3. (B) FEN1 knockdown induced DNA damage and enhanced the activation of the p53 pathway in A549 cells. (C) Overexpression of FEN1 prevented the cell from undergoing cisplatin‐induced apoptosis (5 μm, 72 h) in A549 cells. (D) Representative micrographs of H&E staining, IHC staining (caspase‐3, 53BP1, γ‐H2AX), and TUNEL assay of tumor samples. Scale bars, 250 μm.

The P53 pathway is the most common mechanism of apoptosis. Under stress, P53 is activated by postphosphorylation and acts as a transcription factor that activates the expression of genes involved in apoptosis. Although A549 has a mutant EGFR and elevated EGF pathway activity, it has a wild‐type P53 gene. We tested whether FEN1 deficiency‐induced apoptosis was p53 dependent. We showed that both p53 and the phosphorylated form of p53 have been induced by cisplatin (Fig. 7A), indicating that the p53 pathway was activated by cisplatin. As a downstream event of p53 activation, the apoptosis indicator, cleaved caspase‐3, was also up‐regulated in A549 cells after cisplatin exposure. Moreover, the DNA damage level, as indicated by γ‐H2AX, was correlated with the up‐regulation of the apoptosis indicator and up‐regulated by cisplatin (Fig. 7A).

The correlated up‐regulation between γ‐H2AX and cleavage caspase‐3 promoted us to test whether DNA damage could induce an apoptotic response. In order to figure this out, we treated A549 cells with FEN1 siRNA and the apoptotic responses in A549 cells were measured. As expected, FEN1 knockdown increased the level of γ‐H2AX. Simultaneously, the expression of phosphorylated P53 and cleaved caspase‐3 was also up‐regulated by FEN1 knockdown, similar to cisplatin treatment (Fig. 7B). These data suggested that DNA damage resulted from FEN1 knockdown and could induce p53‐dependent apoptosis. To further confirm the role of FEN1 and DNA damage level in cisplatin‐induced apoptosis, we determined the cellular response to cisplatin in A549 cells with FEN1 overexpression. As shown in Fig. 7C, overexpression of FEN1 reduced cisplatin‐induced DNA damage. The cisplatin‐induced p53 activation and cleaved caspase‐3 formation were also reduced. This observation suggested that FEN1 protected cells from cisplatin‐induced apoptosis by reducing DNA damage in cells.

To further study the mechanisms of FEN1 inhibition‐induced apoptosis *in vivo*, the tumor in Fig. 6C was subjected to IHC assays. As shown in Fig. 7D, the combination of cisplatin and FEN1 inhibitor showed greater collaborative effects on cell apoptosis compared with caspase‐3‐ and TUNEL‐positive cell treatments. In addition, and consistent with previous findings, treatment with the combination of FEN1 inhibitor and cisplatin resulted in more DSBs in tumors, as indicated by γ‐H2AX and 53BP1 staining, than treatment with FEN1 inhibitor or cisplatin alone. Taken together, the above results suggested that FEN1 down‐regulation or inhibition activated the p53‐mediated intrinsic pathway of apoptosis.

## 4 Discussion

FEN1 has been reported to be overexpressed in lung cancer, testis and brain tumors, and altered FEN1 expression might impact the therapeutic response (Nikolova *et al*., 2009). In prostate cancer, FEN1 is overexpressed and is associated with a high Gleason score, which suggests that FEN1 might be a potential marker for prostate cancer diagnosis and therapy (Lam *et al*., 2006; Posadas *et al*., 2009). Mice carrying with FEN1 E160D mutation were predisposed to autoimmunity, chronic inflammation, and cancers, which results in the initiation and progression of cancer (Zheng *et al*., 2007b). FEN1 mutations that specifically disrupt the PCNA interaction have been reported to cause aneuploidy‐associated cancer progression (Zheng *et al*., 2007a). It was found that the FEN1‐69GG genotypes were significantly correlated with increased risk for developing breast cancer, which highlights FEN1 as an important gene in human breast carcinogenesis (Lv *et al*., 2014). Genomic and protein expression analyses revealed FEN1 as a key biomarker in breast and ovarian epithelial cancers, in which FEN1 overexpression is associated with high grade, high stage, and poor survival (Abdel‐Fatah *et al*., 2014). Our team identified the FEN1 mutation in colorectal cancer cells and evaluated its function in cancer progression (Sun *et al*., 2017). Based on these reports, we speculate that FEN1 could be used as a promising cancer diagnostic biomarker.

FEN1 plays important roles in the removal of the RNA primer during Okazaki fragment maturation and in the removal of flap structures in LP BER (Balakrishnan and Bambara, 2013). Thus, FEN1 has dual functions in DNA replication and repair. FEN1 has been suggested to be required in fast‐dividing cells, such as cancer cells. The use of FEN1 as a key biomarker in breast, ovarian, and gastric cancers has been attempted (Abdel‐Fatah *et al*., 2014; Wang *et al*., 2014). In this study, we found that FEN1 overexpression is associated with cancer progression, while it is inversely correlated with survivorship in non‐small‐cell lung cancer. Although the correlation between FEN1 expression and tumor progression had been demonstrated, further study was required to clarify whether the overexpression of FEN1 was a cause or a result of tumor progression. We hypothesized that the inhibition of FEN1 could suppress cancer growth by blocking DNA synthesis. Indeed, siRNA‐mediated down‐regulation of FEN1 in A549 cells slowed cell proliferation, leading to the accumulation of unrepaired DSBs and a higher percentage of sub‐G1 cells. Inhibition of FEN1 by a small‐molecule inhibitor also yielded similar results. Meanwhile, and consistent with the previously reported findings, the overexpression of FEN1 promoted cell proliferation, colony formation and induced tumorigenesis. These data indicated that the overexpression of FEN1 was a trigger for tumor initiation and progression rather than a reflection of rapid cell division.

In addition to its role in DNA replication, increased FEN1 expression may also be a response to severe DNA damage in cancer cells. FEN1 has been reported to be induced by genotoxic stress in various cells (Markus Christmann *et al*., 2005; Wang *et al*., 2015). Consistent with this finding, we found that FEN1 could be induced by cisplatin (Fig. S1). Moreover, we demonstrated that FEN1 overexpression protected NSCLC against cisplatin (Fig. 3A). NSCLC is one of the deadliest human diseases, and cisplatin has been widely used as a chemotherapeutic drug for the treatment of NSCLC. However, long‐term treatment of NSCLC by cisplatin will induce drug resistance of NSCLC to cisplatin. Because the intrastrand crosslink is the major lesion caused by cisplatin, it is primarily repaired via the NER system (Masters and Koberle, 2003). In addition, the homologous recombination repair (HR) that allows error‐free repair of the double‐strand breaks caused by the excision of cisplatin–DNA adducts has been implicated in the repair of cisplatin‐induced DNA damage (Borst *et al*., 2008). The MMR system has also been reported to recognize cisplatin‐induced DNA damage (Sedletska *et al*., 2007).

The direct roles of FEN1 in the repair of cisplatin‐induced DNA lesions have not yet been reported. However, previous reports have demonstrated that the down‐regulation of FEN1 can increase the sensitivity of cisplatin in LN308 glioma cells and SGC‐7901 gastric cancer cell (Nikolova *et al*., 2009; Xie *et al*., 2016), implying the involvement of FEN1 in the repair of cisplatin‐induced DNA damage. Indeed, besides its role in BER, FEN1 has also been reported to be involved in MMR (Johnson *et al*., 1995; Liu *et al*., 2015), NER (Mocquet *et al*., 2008; Shivji *et al*., 1995), and HR (Fehrmann *et al*., 2015; Shivji *et al*., 1995). Based on the reports above, we believed that FEN1 expression might impact the therapeutic response of cisplatin. We further speculated that a high FEN1 expression level in NSCLC contributed to intrinsic or acquired drug resistance. Therefore, we altered the FEN1 level in A549 cells. The results showed that FEN1‐deficient cells were more sensitive to cisplatin treatment, leading to accumulation of unrepaired DSBs in cells. However, overexpression of FEN1 protected cells from apoptosis induced by cisplatin. These data indicated that FEN1 was a determinant of cisplatin resistance in non‐small‐cell lung cancer.

The dual function of FEN1 in DNA replication and repair makes it an ideal target for cancer therapy. Inhibiting FEN1 in cancer cells not only suppressed cancer progression but also enhanced the toxicity of DNA damage‐inducing agents. The toxicity induced by FEN1 inhibition or down‐regulation were the combined results of failures in both DNA replication and repair. Due to higher rates of replication compared to noncancerous cells, cancer cells accumulate and tend to have more innate DNA damage. Moreover, cancer cells are usually defective in cell cycle checkpoints and have shorter repair times. For these reasons, the inhibition of FEN1 has more severe impacts on cancer cells than on the surrounding normal tissues. The efficacy of FEN1 inhibition could be further enhanced by combining FEN1 inhibition with DNA damage‐inducing agents, such as cisplatin.

Taken together, our findings implicated FEN1 in DNA replication and repair as a mechanism of lung cancer development and cancer drug resistance. In this study, we presented evidence that FEN1 was overexpressed in lung cancer and promoted tumor progression *in vitro* and *in vivo*. Down‐regulation by siRNA or inhibition FEN1 activity by small‐molecule inhibitor suppressed cell proliferation and sensitized lung cancer cells to cisplatin. Using a tumor mouse model, we showed that the inhibition of FEN1 impeded the progression of tumor growth by activating intrinsic pathway of apoptosis, thereby enhancing the animal's lifespan. These data suggested that targeting FEN1 could be a potential strategy for the treatment of lung cancer.

## Author contributions

ZGG, LFH, and LBL conceived and designed the project and performed most of the experiments. FJ, ZHZ, HYC, and LJL acquired clinical data and did statistics analysis. CSK, FYP, and ZGH wrote the manuscript. HZ, HY, and HW did animal experiments and western blotting experiments. HFS helped with cell culture. YLZ and JZ contributed to the data processing and manuscript revision.

## Supporting information


**Fig. S1.** FEN1 expression was elevated by cisplatin treatment.
**Fig. S2.** Chemical structure of compound 20.
**Fig. S3**. FEN1 expression level in different kind of cell lines. FEN1 is overexpressed in NSCLC cell line A549.
**Fig. S4.** (A) A549‐Cisplatin resistance cell line was cultured in 2 μg·mL^−1^ cisplatin containing medium. (B) Cell survival between A549 normal cell line and cisplatin resistance cell line when treated with 2 μg·mL^−1^ cisplatin. (C) Cell survival rate of A549‐cisplatin resistance cell line when treated with FEN1 inhibitor C20.
**Table S1.** Associations between FEN1 expression and clinical/histological parameters in lung cancer patients.
