# FEN1 promotes tumor progression and confers cisplatin resistance in non‐small‐cell lung cancer

**DOI:** 10.1002/1878-0261.12118

**Published:** 2017-09-01

**Authors:** Lingfeng He, Libo Luo, Hong Zhu, Huan Yang, Yilan Zhang, Huan Wu, Hongfang Sun, Feng Jiang, Chandra S. Kathera, Lingjie Liu, Ziheng Zhuang, Haoyan Chen, Feiyan Pan, Zhigang Hu, Jing Zhang, Zhigang Guo

Volume 11, Issue 6, pages 640–654. First published: 12 May 2017

The authors of this article have supplied the following correction.

Figure 1E was mistakenly replaced by Fig. 1G of He *et al*. (2016) during the final assembly of the figures for review. The corrected figure is provided below. The authors apologize for the error.

**Figure 1:**
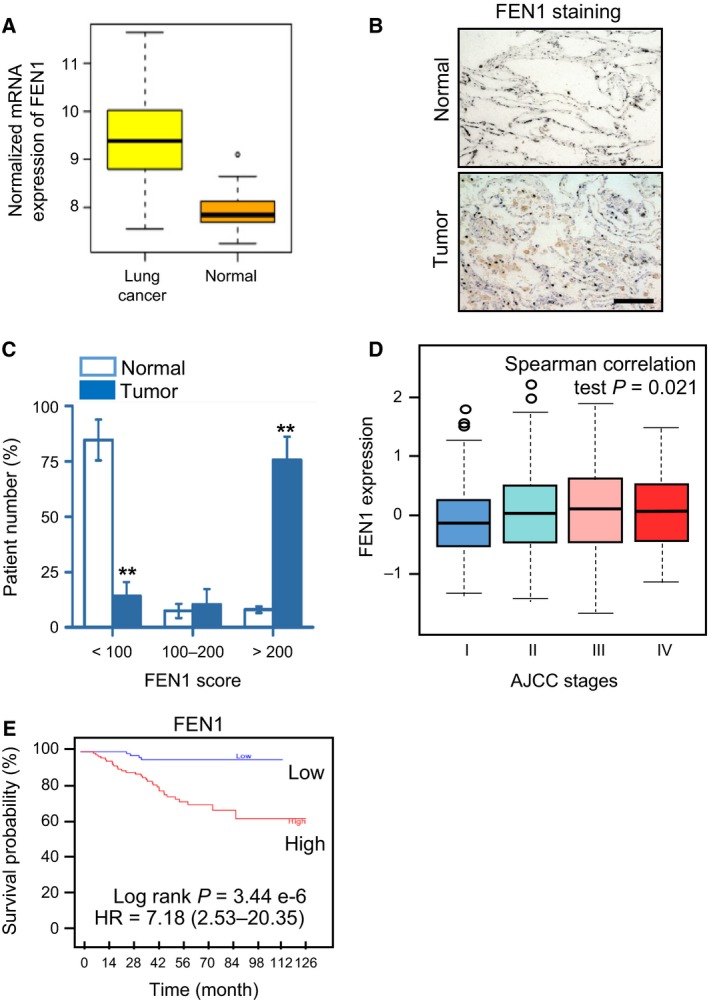
FEN1 overexpression was associated with lung cancer. (A) FEN1 expression analysis based on TCGA dataset showed that FEN1 mRNA levels were higher in lung cancer tissue than in normal tissue (*P < 0.01 vs control group). (B) FEN1 displayed significantly stronger staining (brown) in tumor specimens from clinical patients than from healthy counterparts. Immunohistochemistry was performed on formalin‐fixed and paraffin‐embedded tissues using antibodies against FEN1. Original magnification, ×400. Scale bars, 250 μm. (C). Number of patients’ samples with FEN1 score > 200 was significantly higher in tumors than in normal tissues (**P < 0.01 vs control group). (D) FEN1 expression was correlated with the clinical stage of lung cancer. Spearman's correlation test, *P* = 0.021. (E) Kaplan–Meier analysis of survival of patients with lung cancer. Log rank *P* = 3.44 e‐6.
