# Supplementary material for: FEN1 promotes tumor progression and confers cisplatin resistance in non‐small‐cell lung cancer
Source: Mol Oncol. 2017 May 12;11(6):640–54. doi: 10.1002/1878-0261.12058 (PMC5467497; doi:10.1002/1878-0261.12058)
Supplement: Supplementary file 1 — Fig. S1. FEN1 expression was elevated by cisplatin treatment. Fig. S2. Chemical structure of compound 20. Fig. S3. FEN1 expression level in different kind of cell lines. FEN1 is overexpressed in NSCLC cell line A549. Fig. S4. (A) A549‐Cisplatin resistance cell line was cultured in 2 μg·mL−1 cisplatin containing medium. (B) Cell survival between A549 normal cell line and cisplatin resistance cell line when treated with 2 μg·mL−1 cisplatin. (C) Cell survival rate of A549‐cisplatin resistance cell line when treated with FEN1 inhibitor C20. Table S1. Associations between FEN1 expression and clinical/histological parameters in lung cancer patients. [file MOL2-11-640-s001.pptx]

## Slide 1
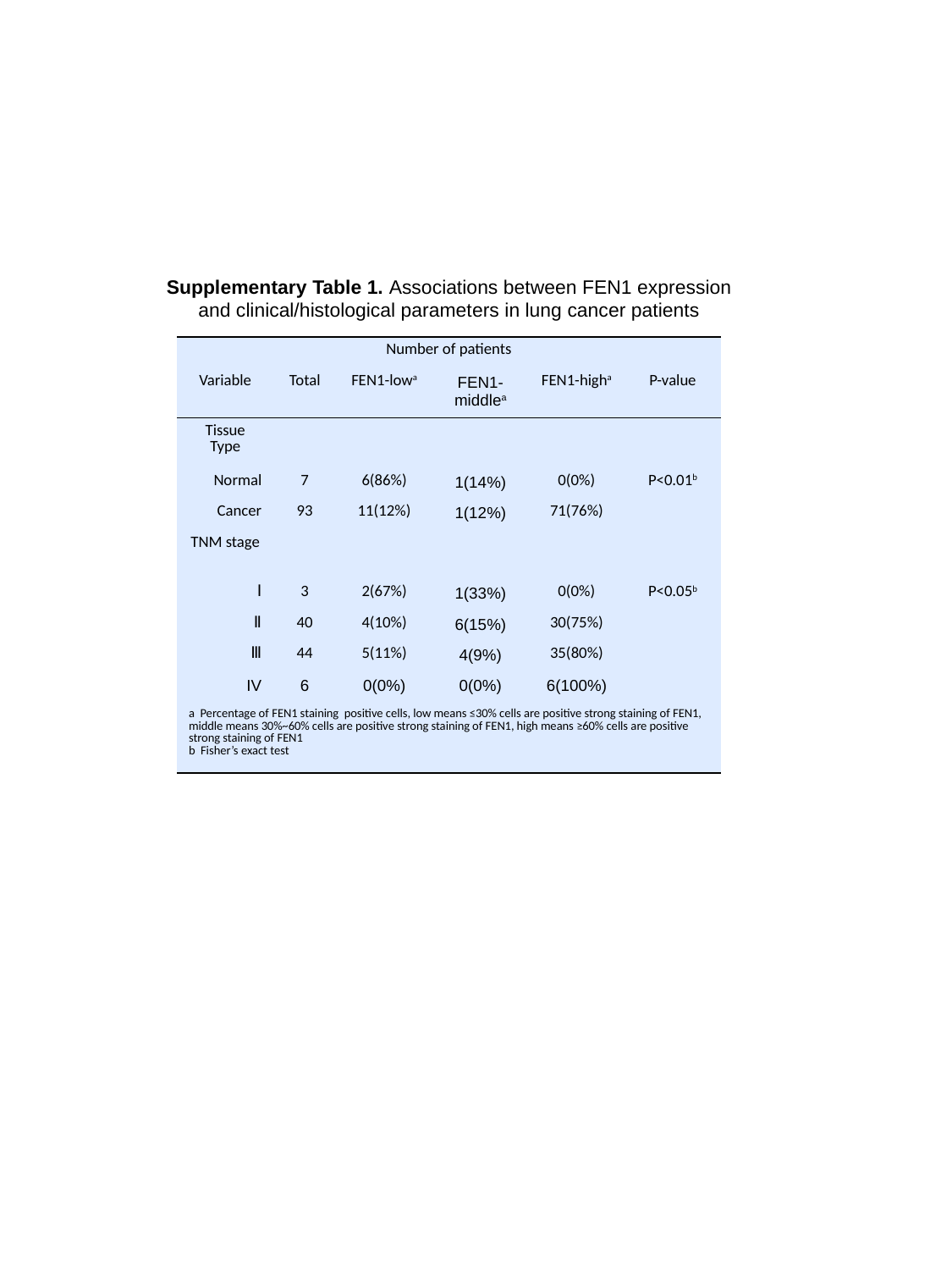

Supplementary Table 1. Associations between FEN1 expression and clinical/histological parameters in lung cancer patients
| | Number of patients | | | | |
| --- | --- | --- | --- | --- | --- |
| Variable | Total | FEN1-lowa | FEN1-middlea | FEN1-higha | P-value |
| Tissue Type | | | | | |
| Normal | 7 | 6(86%) | 1(14%) | 0(0%) | P<0.01b |
| Cancer | 93 | 11(12%) | 1(12%) | 71(76%) | |
| TNM stage | | | | | |
| Ⅰ | 3 | 2(67%) | 1(33%) | 0(0%) | P<0.05b |
| Ⅱ | 40 | 4(10%) | 6(15%) | 30(75%) | |
| Ⅲ | 44 | 5(11%) | 4(9%) | 35(80%) | |
| IV | 6 | 0(0%) | 0(0%) | 6(100%) | |
| a Percentage of FEN1 staining positive cells, low means ≤30% cells are positive strong staining of FEN1, middle means 30%~60% cells are positive strong staining of FEN1, high means ≥60% cells are positive strong staining of FEN1 b Fisher’s exact test | | | | | |

## Slide 2
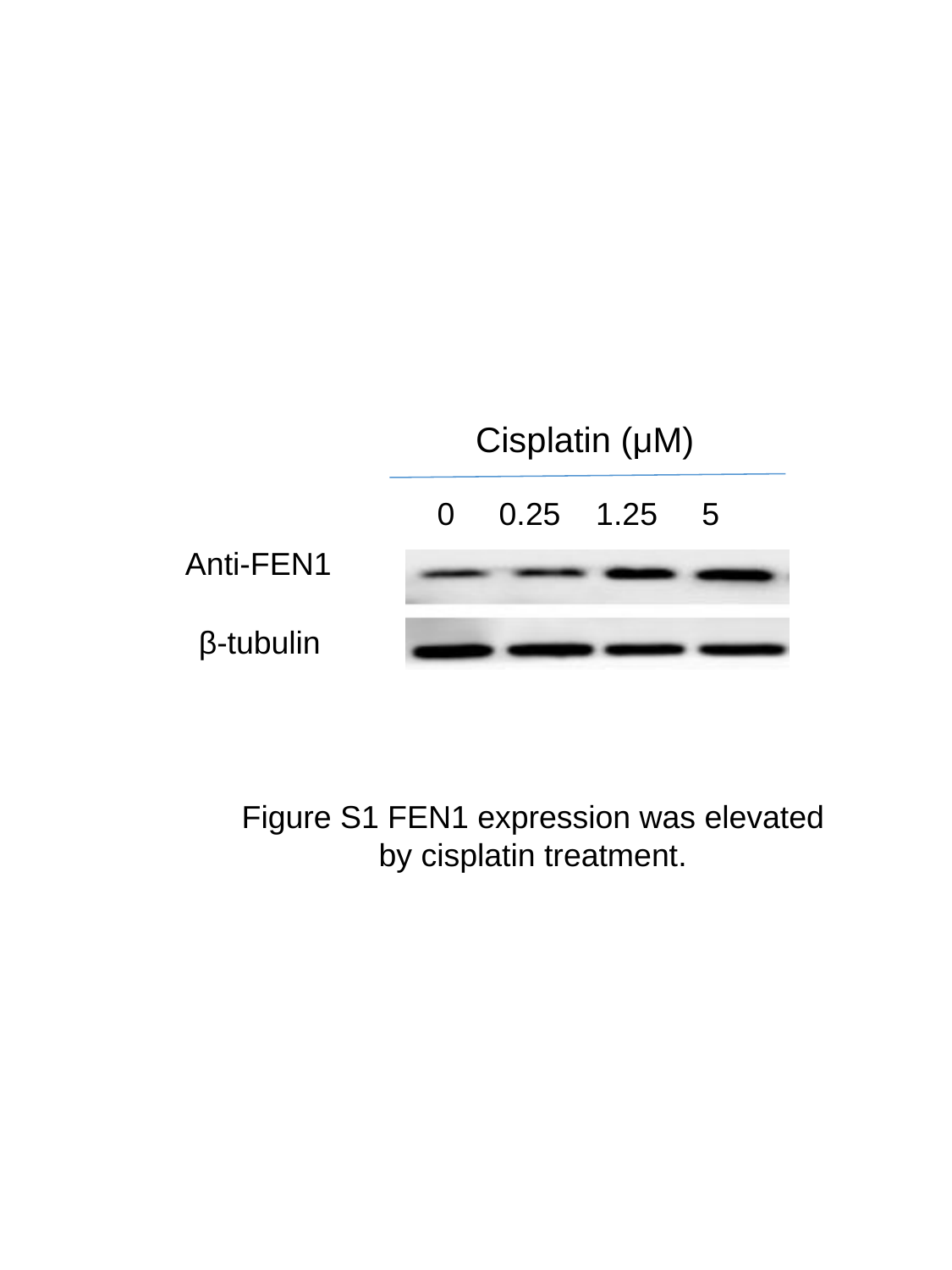

Cisplatin (μM)
 0 0.25 1.25 5
Anti-FEN1
β-tubulin
Figure S1 FEN1 expression was elevated by cisplatin treatment.

## Slide 3
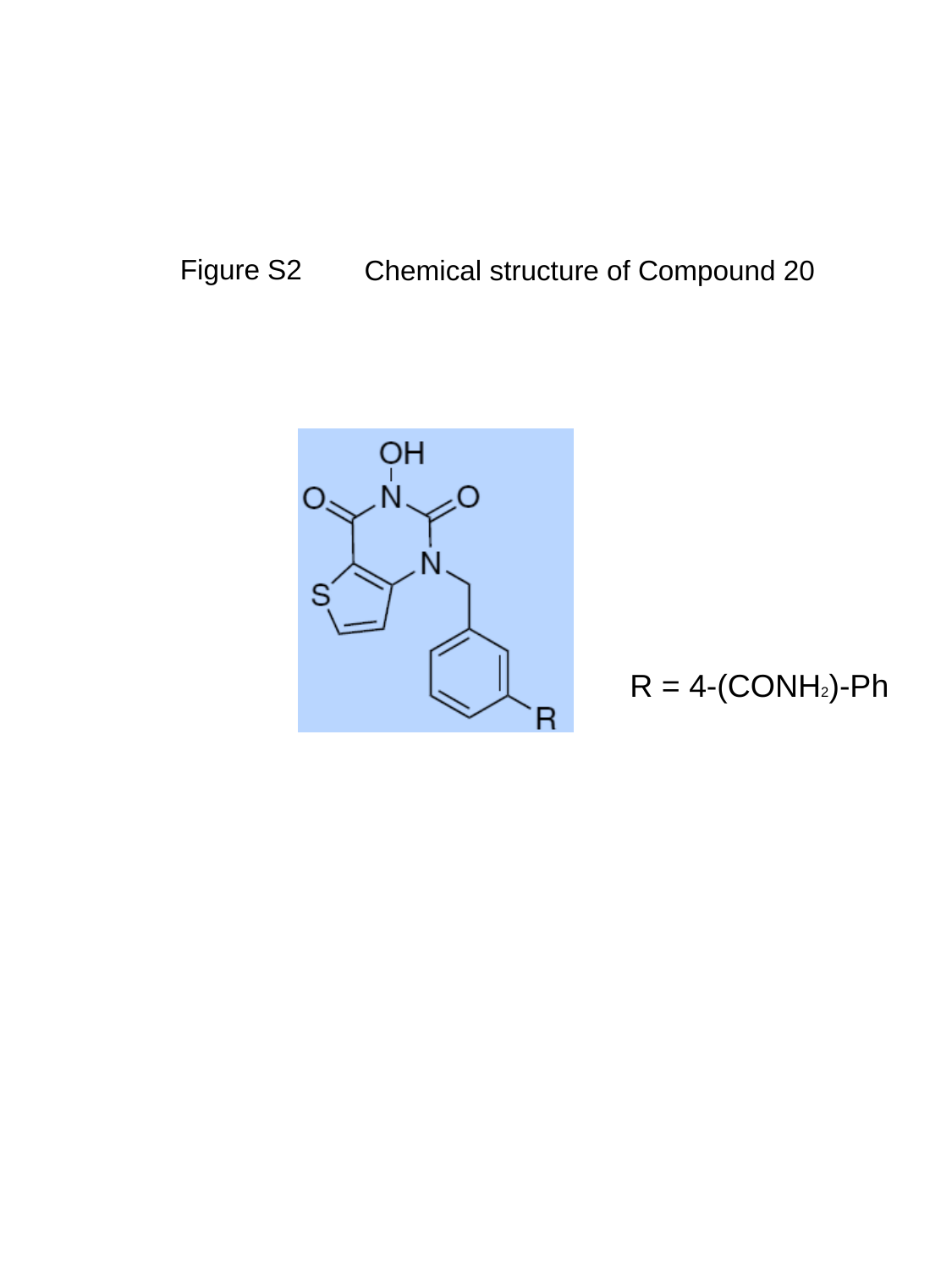

Figure S2
Chemical structure of Compound 20
R = 4-(CONH2)-Ph

## Slide 4
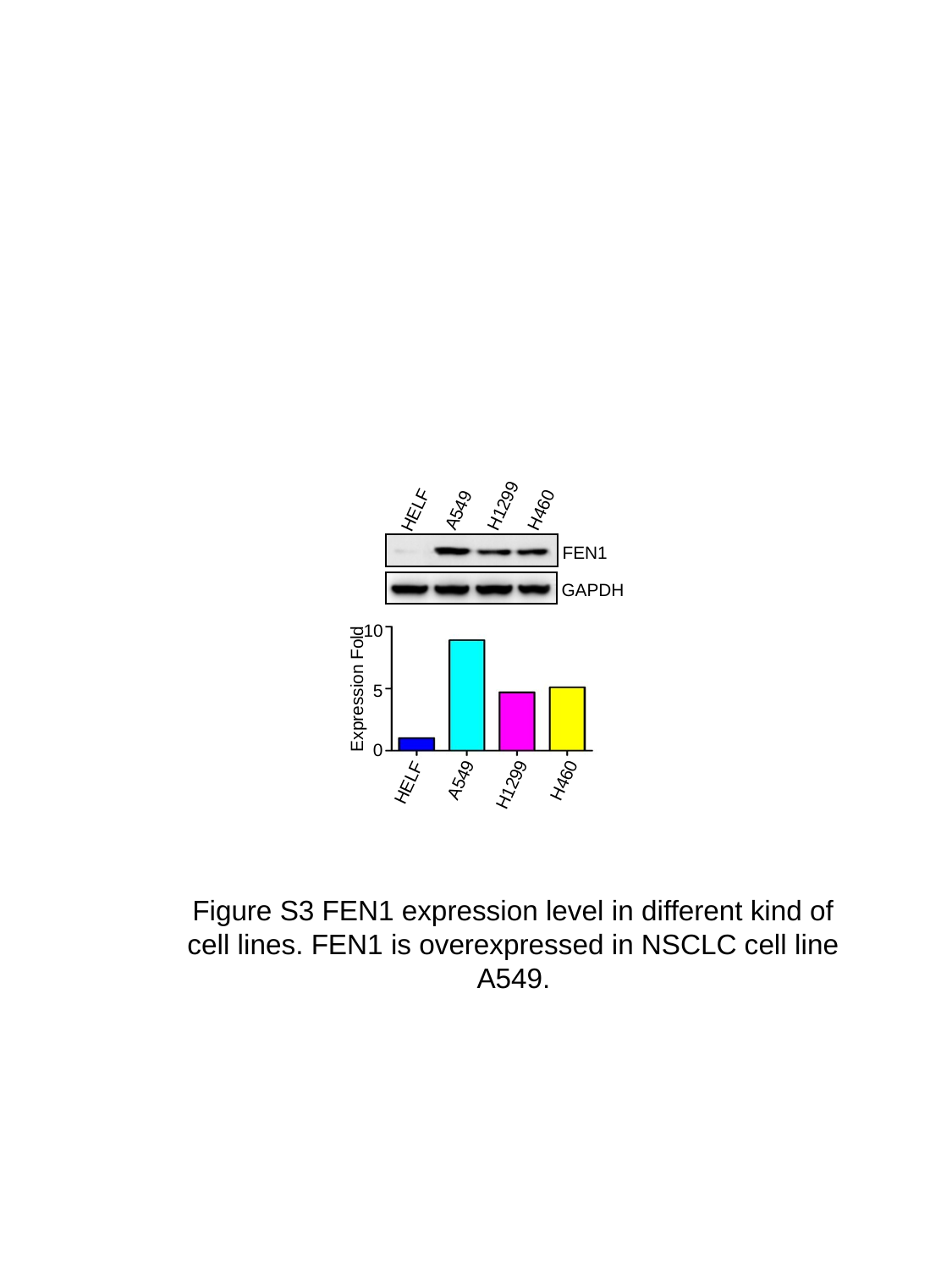

H460
H1299
A549
HELF
FEN1
GAPDH
10
Expression Fold
5
0
A549
H460
H1299
HELF
Figure S3 FEN1 expression level in different kind of cell lines. FEN1 is overexpressed in NSCLC cell line A549.

## Slide 5
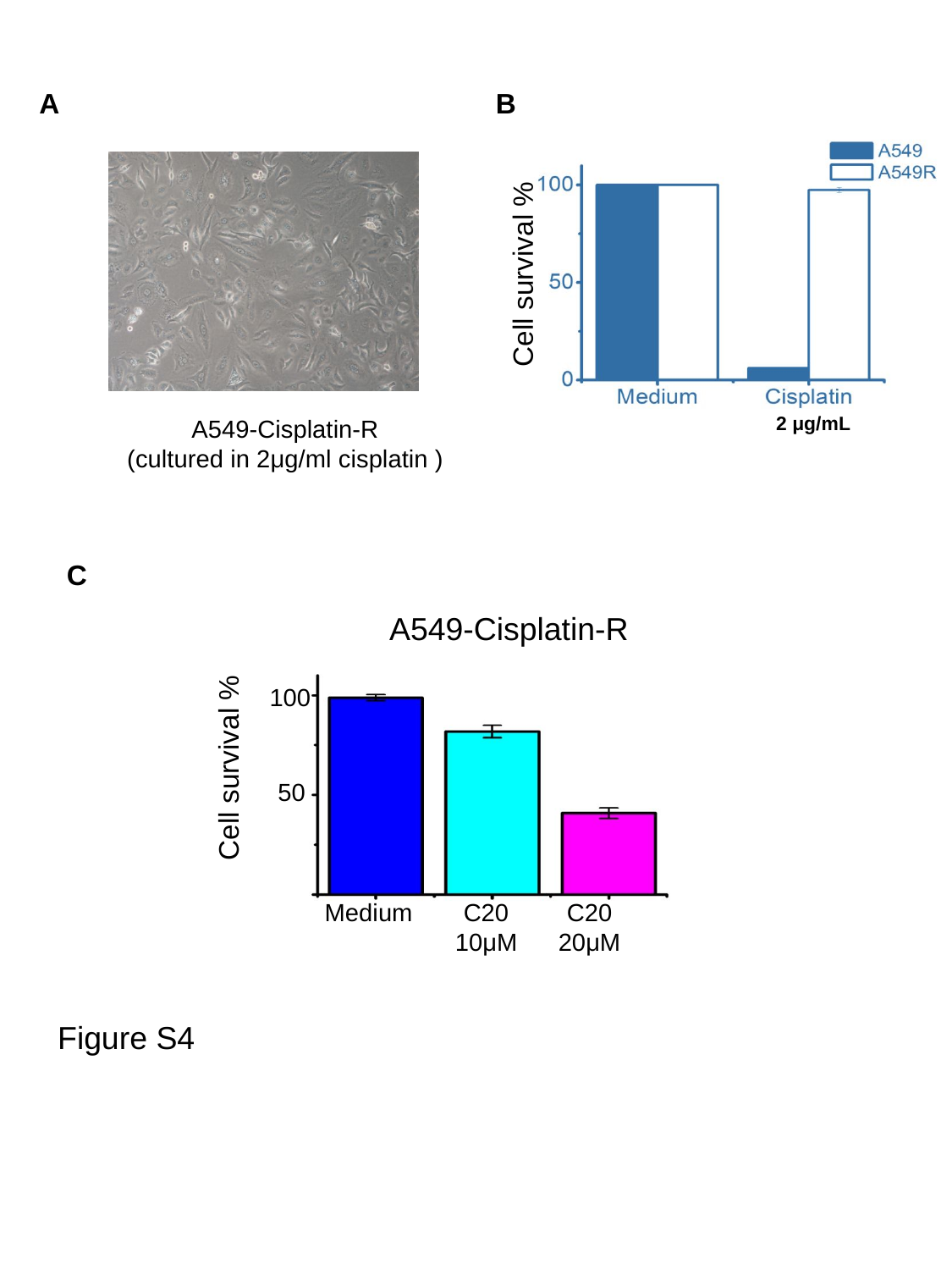

B
A
Cell survival %
2 μg/mL
A549-Cisplatin-R
(cultured in 2μg/ml cisplatin )
C
A549-Cisplatin-R
Cell survival %
100
50
Medium
C20
10μM
C20
20μM
Figure S4
